# Trigeminal neuralgia is associated with increased risk of cerebrovascular disease, myocardial infarction and suicide – a nationwide Swedish study

**DOI:** 10.1186/s10194-026-02368-1

**Published:** 2026-04-18

**Authors:** Erik Ehinger, Erik Kronvall, Erik Uvelius

**Affiliations:** 1 Neurosurgery, Department of Clinical Sciences Lund, Lund University, Lund, Sweden; 2 Department of Neurosurgery, Skåne University Hospital, EA-Blocket Plan 3, Lund, 221 85 Sweden

**Keywords:** Trigeminal neuralgia, Chronic pain, Neuropathic pain, Comorbidity, Mortality, Epidemiology

## Abstract

**Background:**

Trigeminal neuralgia is sometimes referred to as *the suicide disease*, but long-term occurrence of comorbidities and mortality are not well known. The aim of the present study was to investigate whether patients diagnosed with trigeminal neuralgia have an increased risk of morbidity and mortality compared to the general population.

**Methods:**

A nationwide, retrospective cohort study was conducted using Swedish national health and death registers. Adults diagnosed with trigeminal neuralgia between 2001 and 2005 were included. Comorbidities were identified from the time of diagnosis and onwards. Standardised incidence ratios (SIR) and standardised mortality ratios (SMR) were calculated.

**Results:**

The cohort comprised 3531 patients with a median follow-up time of more than 17 years. SIR was significantly elevated for cerebrovascular disease (SIR 1.4–1.8), acute myocardial infarction (SIR 1.3), external causes of injury (SIR 1.5) and intentional self-harm (SIR 2.1). Male patients had a higher risk of myocardial infarction and ischemic stroke, while women had an increased risk of intentional self-harm and unintentional poisoning. No increase was seen for overall mortality, but suicide risk was more than doubled among women (SMR 2.5).

**Conclusion:**

This study highlights the severe impact of trigeminal neuralgia on patients’ lives, supporting the characterisation of trigeminal neuralgia as a true suicide disease. Further studies are warranted to examine causality and to analyse the effects of early treatment, especially surgical intervention.

**Supplementary Information:**

The online version contains supplementary material available at 10.1186/s10194-026-02368-1.

## Background

Trigeminal neuralgia (TN) is a clinically well-defined syndrome of recurrent attacks of orofacial pain triggered by innocuous sensory stimuli [1]. It is estimated that TN afflicts 5.5–12.6/100,000 persons/year with a female predominance [2, 3]. The intense severity of the attacks and the unpredictable course of the affliction are thought to impose considerable psychological stress. In historical accounts, TN has been given the epithet *the suicide disease* due to the unbearable nature of the condition [4]. Other headache syndromes, as well as other diagnoses associated with chronic pain, have been linked to increased rates of suicide [5, 6]. Although depression, anxiety and impaired quality of life have been reported to be more common in patients with TN [7], we have found no studies that confirm an increased risk of suicide in these patients. Furthermore, affective disorders may not be the only health-related consequence of a severe pain condition such as TN; other causes of chronic pain have been linked to an increase in cardiovascular and cerebrovascular morbidity and mortality [8].

We hypothesise that TN influences morbidity and mortality in similar ways as other chronic pain disorders. Therefore, this study aimed to investigate whether patients diagnosed with TN have increased morbidity and mortality from cerebrovascular and cardiovascular disease or from external causes, including intentional self-harm, using data from Swedish national health registries.

## Methods

This was a nationwide, retrospective study based on data from the Swedish National Patient Register and the Swedish National Cause of Death Register, both managed by the Swedish National Board of Health and Welfare. Patients were identified by their unique Swedish national personal identification number for cross-reference between the two registers. The patient register has national coverage and contains information on every hospital admission since 1987, and every outpatient clinic visit to a specialist department since 2001. Primary health care is not included. The Cause of Death Register also has national coverage and information on cause of deaths since 1961. Both registers are reported to have excellent coverage [9, 10].

Adult patients aged ≥18 years diagnosed with TN from 2001 to 2005 were identified in the patient register by the ICD-10 code for TN (G50.0). The start of the inclusion period was selected to also include specialist outpatient visits, which were registered from 2001 in the Swedish National Patient Register. Five full calendar years were selected to include a sufficiently large cohort of TN patients. Individuals who had received a TN diagnosis (ICD-10 code G50.0 or ICD-9 code 350) before 2001 were excluded. Thus, only patients who were first diagnosed with TN during the study period were included.

The occurrence of comorbidities in the patient register was noted from the time of diagnosis of TN until the end of follow-up (December 31, 2022) or death. Comorbidities were selected through a literature review on the potential effects of chronic pain and TN [8, 11–13] and included the stroke subtypes subarachnoid haemorrhage (I60), intracerebral haemorrhage (I61) and ischemic stroke (I63), along with myocardial infarction (I21), injuries from external causes (S00-T98) including poisoning by drugs, medicaments and biological substance (T36-T50) and external causes of morbidity and mortality (V01-Y98) including falls (W00-W19), accidents (V01-X59) and intentional self-harm (X60-X84). Chronic conditions, such as hypertension and depression, often treated by general practitioners, were not included.

Additionally, cause of death data was retrieved from the Swedish National Cause of Death Register for included patients who died prior to the end of follow-up. Overall mortality, as well as deaths due to a cause of death of any of the ICD-10 diagnoses listed above were noted.

The study was approved by the Swedish Ethical Review Authority (reference 2022-01469-01) and by the National Board of Health and Welfare, Stockholm, Sweden.

### Statistical methods

Demographic data are presented as median (interquartile range, IQR). Person-years at risk were calculated from the time of TN diagnosis to event (morbidity or mortality) or the end of follow-up, and stratified according to sex, five-year age group, and calendar year. Calculations were performed using the first event of each calendar year. Data on the occurrence of the aforementioned comorbidities, in addition to mortality in the Swedish general population, were retrieved from the public database of the Swedish National Board of Health and Welfare [14]. The expected number of cases of each comorbidity, as well as the expected number of deaths divided by sex, five-year age group, and calendar year, were calculated and compared with the observed occurrence in the TN cohort and presented as standardised incidence ratio (SIR) or standardised mortality ratio (SMR) with a 95% confidence interval. Additionally, subgroup calculations of SIR and SMR were performed for the initial three years after diagnosis of TN, as well as for male and female patients separately. Statistical analyses were performed using the statistical software R [15].

## Results

In total 3531 patients, 2326 women (66%) and 1205 men (34%), had a primary diagnosis of TN during 2001 to 2005, resulting in a mean incidence of 10/100 000 persons/year based on population data from Statistics Sweden [16]. The median age at diagnosis was 65 (IQR 52–75) years. The follow-up period included 50 916 patient-years with a median follow-up of 17.3 (IQR 9.5–19.5) years.

The occurrence of all comorbidities, including cerebrovascular diseases (SIR 1.4–1.8), acute myocardial infarction (SIR 1.3), and all types of external causes of injury (SIR 1.1–2.1), including intentional self-harm (SIR 2.1), was significantly increased. SIRs for these comorbidities were increased within the first three years after TN diagnosis **(**Table 1**)**.

**Table 1:** Standardised incidence ratio (SIR) of the total patient cohort during follow-up and during the first three years after diagnosis of trigeminal neuralgia

	SIR during the first three years after diagnosis			SIR during the total follow-up period		
	Observed cases	Expected cases	SIR (95% CI)	Observed cases	Expected cases	SIR (95% CI)
Subarachnoid haemorrhage (I60)	9	3	3.5 (1.6–6.7)	23	13	1.8 (1.2–2.8)
Intracerebral haemorrhage (I61)	16	9	1.8 (1.0-2.9)	83	50	1.7 (1.3-2.0)
Cerebral infarction (I63)	103	63	1.6 (1.3-2.0)	457	339	1.4 (1.2–1.5)
Acute myocardial infarction (I21)	96	70	1.4 (1.1–1.7)	424	325	1.3 (1.2–1.4)
Injuries of external causes (S00-T98)	924	508	1.8 (1.7–1.9)	4720	3238	1.5 (1.4–1.5)
Poisoning by drugs, medicaments and biological substance (T36-T50)	33	9	3.8 (2.6–5.3)	116	49	2.4 (2.0-2.8)
External causes of morbidity and mortality (V01-Y98)	688	514	1.3 (1.2–1.4)	3850	3603	1.1 (1.0-1.1)
Falls (W00-W19)	513	319	1.6 (1.5–1.8)	2880	2226	1.3 (1.3–1.3)
Accidents (V01-X59)	570	397	1.4 (1.3–1.6)	3109	2697	1.2 (1.1–1.2)
Intentional self-harm (X60-X84)	21	7	2.9 (1.8–4.5)	74	35	2.1 (1.7–2.7)

SIR-values differed some between men and women. Male patients with TN more often had ischemic stroke (SIR 1.3), acute myocardial infarction (SIR 1.5), injuries of external causes (SIR 1.4), poisonings (SIR 1.9) and accidental falls (SIR 1.2), but had no increase in intentional self-harm. The same comorbidities, as well as subarachnoid haemorrhage (SIR 6.9), were all increased even during the first three years after diagnosis. Females with TN also more often had ischemic stroke (SIR 1.4), myocardial infarction (SIR 1.2), injuries of external causes (SIR 1.5), poisonings (SIR 2.6), and accidental falls (SIR 1.3). Additionally, they had an increase in intracerebral haemorrhages (SIR 1.9), general accidents (SIR 1.2), and intentional self-harm (SIR 2.6). In females, all comorbidities, except myocardial infarction, occurred more frequently within the first three years after diagnosis **(**Table 2 and Supplemental Table 1).

**Table 2:** Standardised incidence ratio (SIR) during the follow-up period divided by sex

	SIR during total follow-up period					
	Male patients			Female patients		
	Observed cases	Expected cases	SIR (95% CI)	Observed cases	Expected cases	SIR (95% CI)
Subarachnoid haemorrhage (I60)	10	3	2.9 (1.4–5.3)	13	9	1.4 (0.8–2.5)
Intracerebral haemorrhage (I61)	25	19	1.3 (0.8–1.9)	58	31	1.9 (1.4–2.4)
Cerebral infarction (I63)	165	124	1.3 (1.1–1.6)	292	215	1.4 (1.2–1.5)
Acute myocardial infarction (I21)	217	146	1.5 (1.3–1.7)	207	178	1.2 (1.0-1.3)
Injuries of external causes (S00-T98)	1344	976	1.4 (1.3–1.5)	3376	2262	1.5 (1.4–1.5)
Poisoning by drugs, medicaments and biological substance (T36-T50)	24	13	1.9 (1.2–2.8)	92	36	2.6 (2.1–3.1)
External causes of morbidity and mortality (V01-Y98)	1050	1106	1.0 (0.9-1.0)	2800	2497	1.1 (1.1–1.2)
Falls (W00-W19)	701	566	1.2 (1.2–1.3)	2179	1660	1.3 (1.3–1.4)
Accidents (V01-X59)	771	782	1.0 (0.9–1.1)	2338	1915	1.2 (1.2–1.3)
Intentional self-harm (X60-X84)	11	10	1.1 (0.5–1.9)	63	25	2.6 (2.0-3.3)

During follow-up 1774 patients died, most often due to diseases of the circulatory system (37%, *n* = 664) and tumours (25%, *n* = 443). The overall SMR was lower than expected (0.9). The increased occurrence of acute myocardial infarction did not translate into increased mortality, nor did the increased incidence of external causes of morbidity and mortality, except for intentional self-harm. The occurrence of suicide among patients diagnosed with TN (*n* = 14) was almost doubled compared to the expected value (*n* = 8), resulting in an SMR of 1.8. The same SMR pattern was also seen within the first three years after diagnosis, except no increase in suicide. SMRs for the entire patient cohort are presented in Table 3.

**Table 3:** Standardised mortality ratio (SMR) of the total patient cohort during the follow-up and during the first three years after diagnosis of trigeminal neuralgia

	SMR during the first three years after diagnosis			SMR during the total follow-up period		
	Observed cases	Expected cases	SMR (95% CI)	Observed cases	Expected cases	SMR (95% CI)
Overall mortality	279	329	0.9 (0.8-1.0)	1174	1912	0.9 (0.9-1.0)
Acute myocardial infarction	32	29	1.1 (0.8–1.6)	121	126	1.0 (0.8–1.2)
Cerebrovascular diseases	21	28	0.7 (0.5–1.1)	97	150	0.7 (0.5–0.8)
External causes of morbidity and mortality	11	10	1.2 (0.6–2.1)	69	61	1.1 (0.9–1.4)
Intentional self-harm	2	2	1.3 (0.1–4.6)	14	8	1.8 (1.0-3.1)

As with SIRs, there were differences in SMRs between male and female patients. Male patients with TN had the same frequency of cause of mortality as the general population, whereas the reduction in overall SMR and death due to cerebrovascular disease, in addition to the increase in suicide, stemmed from female patients. The SMR for suicide among female patients was 2.5. Regardless of sex, suicide was not increased during the early years after diagnosis. 86% of suicides occurred more than three years after the diagnosis of TN. SMRs divided by sex are presented in Table 4.

**Table 4:** Standardised mortality ratio (SMR) during the follow-up period divided by sex

	SMR during total follow-up period					
	Male patients			Female patients		
	Observed cases	Expected cases	SMR (95% CI)	Observed cases	Expected cases	SMR (95% CI)
Overall mortality	627	655	1.0 (0.9-1.0)	1147	1257	0.9 (0.9-1.0)
Acute myocardial infarction	59	53	1.1 (0.8–1.4)	62	72	0.9 (0.7–1.1)
Cerebrovascular diseases	37	46	0.8 (0.6–1.1)	60	104	0.6 (0.4–0.7)
External causes of morbidity and mortality	30	26	1.1 (0.8–1.6)	39	35	1.1 (0.8–1.5)
Intentional self-harm	6	4	1.4 (0.5-3.0)	8	3	2.5 (1.1-5.0)

## Discussion

The present study, including over 3500 TN patients with a median follow-up time of over 17 years, shows that female TN patients have an increased suicide risk compared to the general population. The results also show an increased incidence of non-fatal self-destructive injuries, intoxications, accidents, and falls in TN patients. The occurrence of cerebrovascular disease and myocardial infarction is increased. Interestingly, these findings did not translate into an increased mortality.

Trigeminal neuralgia is a chronic pain syndrome [17], often described as one of the most painful conditions that affects humans. The intermittent and unpredictable pain severely reduces quality of life [18]. Although there is evidence of an increased risk of suicide in patients with chronic pain [11] and the fact that TN is often referred to as *the suicide disease* in textbooks [4], to the best of our knowledge, no previous study has specifically examined the occurrence of suicide and self-harm in patients with TN. Additionally, through various pathophysiologic mechanisms, chronic pain may independently contribute to a broader systemic morbidity, thereby implicating chronic pain in the development of cardiovascular disease, metabolic syndrome and cognitive decline [12, 19, 20]. Accordingly, studies have shown association between chronic pain and increased risk of cerebrovascular disease and ischemic heart disease [8]. The association between TN and these comorbidities remains less studied. This paper aimed to address these knowledge gaps. Several factors that could explain our results are discussed.

### Cardiovascular disease

We found elevated SIRs for both stroke (SIR 1.4–1.8) and acute myocardial infarction (SIR 1.3) among patients with TN. This is consistent with Rönnegård et al. [8], who reported that patients with localised chronic pain had a significantly increased hazard ratio of stroke and myocardial infarction compared to controls. Also, Worm et al. [21] demonstrated an increased risk of cerebral infarction after TN diagnosis, which is in line with our findings. There may be several pathophysiological links between TN and cardiovascular disease. The neurovascular conflict seen in classic TN can in part be attributed to systemic hypertension and atheromatosis, leading to arterial tortuosity and stiffness [22]. Furthermore, antiepileptic drugs (AEDs), the mainstay medical treatment for TN, have been associated with increased cardiovascular and cerebrovascular risk [23].

### Suicide and self-harm

Chronic pain is associated with an increased risk of suicide [6, 11]. A recent study shows that both attempted suicide and completed suicide are more common in primary headache patients [24]. In an interview study on physicians with expertise in headaches, Trejo-Gabriel-Galan et al. found that TN was an uncommon cause of completed suicide among headache patients. Still, TN was the cause of a fourth of all headache-associated suicide attempts [5]. Additionally, Fishbein et al. reported that anxiety, depression, and thoughts of suicide are common in TN patients [25].

It has been shown that individuals with epilepsy, females in particular, have a higher risk of completed suicide than the general population [26]. AEDs have been scrutinised for their potential link to suicidality. The United States Food and Drug Administration issued a blanket warning on increased suicidality with these drugs based on a meta-analysis including, among others, patients treated with AEDs due to chronic pain [27]. The analysis showed an overall 1.8-fold increase in suicidal behaviour in patients treated with AEDs, though no significant increase was noted in patients treated for chronic pain. Additionally, a nationwide Danish study did not find increased suicidality among patients treated with the AEDs most often used in TN, carbamazepine and gabapentin [28].

In the present cohort, the increased occurrence of completed suicide was not evident during the first three years from diagnosis but emerged with longer follow-up time. The delayed increase in suicide risk may be explained by the hypothesis that chronic pain exerts cumulative effects on mental and physical health over time. Also, chronic long-term health conditions, such as TN, may predispose to psychopathological decompensation [29]. As detrimental factors accumulate and interact, their combined impact may amplify over time, increasing the patient’s vulnerability to suicidal tendencies [4]. Although mortality due to suicide is significantly increased in our cohort, it should be emphasised that the absolute number of suicides is low. However, our finding that female TN patients had an increased occurrence of completed suicide and intentional self-harm emphasises the importance of a multidisciplinary approach and screening for psychiatric illness in TN patients.

### External causes of injury

The present study shows an increased frequency of injuries of external causes, including intoxications, accidents and falls among both male and female TN patients. This was seen already during the first three years after diagnosis. Side effects of AEDs used in TN treatment, including dizziness, fatigue and confusion, could add to the risk of accidents and falls [30, 31]. In addition, gabapentin has been related to an increased occurrence of injuries, whereas pregabalin, in the same paper, was associated with suicidal behaviour, unintentional overdoses, injuries and road traffic accidents [30]. In a study on the comorbidities of TN, Worm et al. noted an increased occurrence of delirium and Alzheimer’s disease, likely adding to the risk of accidents and external injuries [21]. There is also evidence suggesting that patients with chronic pain have difficulties in road traffic, with chronic pain as a distractor, which could also increase the risk of accidents [32]. We have found no other study specifically addressing TN in this context.

### Mortality

Several studies have examined the relationship between chronic pain and excess mortality, though findings have been conflicting [33, 34]. Increased mortality has, inconsistently, been shown to be caused by cardiovascular disease, cancer, liver disease, suicide and accidents [33, 35]. Overall, evidence suggests that chronic pain may influence mortality through multifactorial pathways involving lifestyle factors, socio-economic status, psychiatric conditions, and somatic comorbidities [33]. Several studies identify pain significantly affecting patients’ everyday life and causing physical limitations as the main cause of pain-related overall mortality [35].

In our study, the increased incidence of cardiovascular disease did not translate into higher cardiovascular mortality. Similar patterns have been observed in research specifically on localised chronic pain, where myocardial infarction and stroke risks are elevated, but cardiovascular mortality remains unchanged [8]. In contrast, widespread chronic pain has been linked to higher cardiovascular mortality [8]. Elevated all-cause mortality in severe chronic pain, particularly among younger individuals has also been reported [36].

### Limitations

Although our study cohort had demographics similar to those previously reported, indicating that the data provided by the national registry are representative, there are qualitative data limitations to the registry-based approach. The data were limited to hospital care. Patients with a primary care diagnosis of TN were omitted. In a study that also included primary care, Svedung Wettervik et al. reported that most TN diagnoses are made in-hospital. The proportion of patients with no hospital contact due to TN is unknown, but patients with severe and persistent symptoms would likely be referred to a neurologist, which may limit the number of missing cases. Svedung Wettervik et al. also found that a significant share of TN diagnoses was inaccurate after the authors conducted a thorough review of medical records [2]. Such case validation to limit misclassification and false-positive diagnoses is not possible in a registry-based study. It is likely that a larger share of misdiagnoses is made in a primary care setting rather than in hospital care, where neurologists diagnose most patients. These diagnostic limitations also apply to the studied comorbidities. Additionally, there is often a significant delay between symptom onset and diagnosis. While the mean diagnostic delay is around one year, it may extend over many years in a large proportion of the patients [37]. When interpreting our results, it is important to note that we can only consider time from diagnosis, not symptom duration. The registry data allow adjustment for age and sex but do not account for other potential differences, such as smoking, obesity, and other shared risk factors, prior to inclusion. The size of this cohort compensates to some extent for the heterogeneity in the diagnostic accuracy of TN, making the results more generalisable.

In summary, this study has confirmed the suspicion that TN is associated with excess morbidity and suicidality. Several factors could, by themselves or in combination, explain the increased frequency of cardiovascular disease as well as increased morbidity due to external causes and self-harm. However, the aim of the present study was not to assess causality. Further studies are warranted to assess the complex and likely multifactorial cause of elevated morbidity, especially to clarify whether the benefit of early medication is offset by subjecting patients to increased risk of comorbidities. Considering the potential detrimental side effects of AEDs, the effects of surgical intervention for TN are particularly interesting to examine.

## Conclusions

Female TN patients have an increased suicide risk compared to the general population, substantiating the risk of suicide in TN patients. TN is also associated with an increased risk of cardiovascular disease, intoxications, accidents and falls. Except for intentional self-harm, no other comorbidity translates to increased mortality. The findings emphasise the likely need for psychiatric screening in TN patients. Further studies are warranted to examine causality and to analyse the effects of early treatment, especially surgical intervention.

## Supplementary Information

Below is the link to the electronic supplementary material.

Supplementary Material 1

## Data Availability

The datasets used and analysed during the current study are available from the corresponding author on reasonable request.

## Abbreviations

TN: Trigeminal neuralgia
SIR: Standardised incidence ratio
SMR: Standardised mortality ratio
AED: Antiepileptic drug
