# Supplementary material for: Trigeminal neuralgia is associated with increased risk of cerebrovascular disease, myocardial infarction and suicide – a nationwide Swedish study
Source: J Headache Pain. 2026 Apr 18;27(1):117. doi: 10.1186/s10194-026-02368-1 (PMC13101167; doi:10.1186/s10194-026-02368-1)
Supplement: Supplementary file 1 — Supplementary Material 1 [file 10194_2026_2368_MOESM1_ESM.docx]

**Supplemental tables**

| **Supplemental Table 1.** Standardised incidence ratio (SIR) divided by sex during the first three years after diagnosis of trigeminal neuralgia. | | | | | | | |
| --- | --- | --- | --- | --- | --- | --- | --- |
|  | **SIR during the first three years after diagnosis of trigeminal neuralgia** | | | | | | |
|  | **Male patients** | | |  | **Female patients** | | |
|  | **Observed cases** | **Expected cases** | **SIR (95% CI)** |  | **Observed cases** | **Expected cases** | **SIR (95% CI)** |
|  |  |  |  |  |  |  |  |
| Subarachnoid hemorrhage (I60) | 5 | 1 | 6.9 (2.2-16.0) |  | 4 | 2 | 2.2 (0.6-5.7) |
| Intracerebral hemorrhage (I61) | 5 | 4 | 1.4 (0.4-3.2) |  | 11 | 5 | 2.0 (1.0-3.6) |
| Cerebral infarction (I63) | 44 | 24 | 1.8 (1.3-2.5) |  | 59 | 39 | 1.5 (1.2-2.0) |
|  |  |  |  |  |  |  |  |
| Acute myocardial infarction (I21) | 65 | 32 | 2.0 (1.6-2.6) |  | 31 | 38 | 0.8 (0.6-1.2) |
|  |  |  |  |  |  |  |  |
| Injuries of external causes (S00-T98) | 262 | 162 | 1.6 (1.4-1.8) |  | 662 | 347 | 1.9 (1.8-2.1) |
| Poisoning by drugs, medicaments and biological substance (T36-T50) | 6 | 2 | 2.7 (1.0-5.8) |  | 27 | 6 | 4.1 (2.8-6.1) |
|  |  |  |  |  |  |  |  |
| External causes of morbidity and mortality (V01-Y98) | 183 | 165 | 1.1 (1.0-1.3) |  | 505 | 349 | 1.5 (1.3-1.6) |
| Falls (W00-W19) | 118 | 85 | 1.4 (1.2-1.7) |  | 395 | 234 | 1.7 (1.5-1.9) |
| Accidents (V01-X59) | 130 | 123 | 1.1 (0.9-1.3) |  | 440 | 274 | 1.6 (1.5-1.8) |
| Intentional self-harm (X60-X84) | 2 | 2 | 1.0 (0.1-3.5) |  | 19 | 5 | 3.7 (2.2-5.6) |

| **Supplemental Table 2.** Standardised mortality ratio (SMR) divided by sex during the first three years after diagnosis of trigeminal neuralgia. | | | | | | | |
| --- | --- | --- | --- | --- | --- | --- | --- |
|  | **SMR during the first three years after diagnosis of trigeminal neuralgia** | | | | | | |
|  | **Male patients** | | |  | **Female patients** | | |
|  | **Observed cases** | **Expected cases** | **SMR (95% CI)** |  | **Observed cases** | **Expected cases** | **SMR (95% CI)** |
|  |  |  |  |  |  |  |  |
| Overall mortality | 124 | 117 | 1.1 (0.9-1.3) |  | 155 | 213 | 0.7 (0.6-0.9) |
| Acute myocardial infarction | 21 | 13 | 1.6 (1.0-2.5) |  | 11 | 16 | 0.7 (0.4-1.3) |
| Cerebrovascular diseases | 8 | 9 | 0.9 (0.4-1.7) |  | 13 | 19 | 0.7 (0.4-1.2) |
| External causes of morbidity and mortality | 6 | 4 | 1.4 (0.5-3.0) |  | 5 | 19 | 0.7 (0.4-1.2) |
| Intentional self-harm | 1 | 1 | 1.1 (0.0-6.1) |  | 1 | 1 | 1.6 (0.0-8.6) |
